# Image-based camera localization: an overview

**DOI:** 10.1186/s42492-018-0008-z

**Published:** 2018-09-05

**Authors:** Yihong Wu, Fulin Tang, Heping Li

**Affiliations:** National Laboratory of Pattern Recognition, Institute of Automation, Chinese Academy of Sciences, Beijing, China, University of Chinese Academy of Sciences, Beijing, China

**Keywords:** PnP problem, SLAM, Camera localization, Camera pose determination

## Abstract

Virtual reality, augmented reality, robotics, and autonomous driving, have recently attracted much attention from both academic and industrial communities, in which image-based camera localization is a key task. However, there has not been a complete review on image-based camera localization. It is urgent to map this topic to enable individuals enter the field quickly. In this paper, an overview of image-based camera localization is presented. A new and complete classification of image-based camera localization approaches is provided and the related techniques are introduced. Trends for future development are also discussed. This will be useful not only to researchers, but also to engineers and other individuals interested in this field.

## Background

Recently, virtual reality, augmented reality, robotics, autonomous driving etc., in which image-based camera localization is a key task, have attracted much attention from both academic and industrial community. It is urgent to provide an overview of image-based camera localization.

The sensors used for image-based camera localization are cameras. Many types of three-dimensional (3D) cameras have been developed recently. This study considers two-dimensional (2D) cameras. The typically used tool for outdoor localization is GPS, which cannot be used indoors. There are many indoor localization tools including Lidar, Ultra Wide Band (UWB), Wireless Fidelity (WiFi), etc.; among these, using cameras for localization is the most flexible and low cost approach. Autonomous localization and navigation is necessary for a moving robot. To augment reality in images, camera pose determination or localization is needed. To view virtual environments, the corresponding viewing angle is necessary to be computed. Furthermore, cameras are ubiquitous and people carry mobile phones that have cameras every day. Therefore, image-based camera localization has great and widespread applications.

The image features of points, lines, conics, spheres, and angles are used in image-based camera localization; of these, points are most widely used. This study focuses on points.

Image-based camera localization is a broad topic. We attempt to cover related works and give a complete classification for image-based camera localization approaches. However, it is not possible to cover all related works in this paper due to length constraints. Moreover, we cannot provide deep criticism for each cited paper due to space limit for such an extensive topic. Further deep reviews on some active important aspects of image-based camera localization will be given in the future or people interested go to read already existing surveys. There have been excellent reviews on some aspects of image-based camera localization. The most recent ones include the following. Khan and Adnan [1] gave an overview of ego motion estimation, where ego motion requires time intervals between two continuous images to be small enough. Cadena et al. [2] surveyed the current state of simultaneous localization and mapping (SLAM) and considered future directions, in which they reviewed related works including robustness and scalability in long-term mapping, metric and semantic representations for mapping, theoretical performance guarantees, active SLAM, and exploration. Younes et al. [3] specially reviewed keyframe-based monocular SLAM. Piasco et al. [4] provided a survey on visual-based localization from heterogeneous data, where only known environment is considered.

Unlike these studies, this study is unique in that it first maps the whole image-based camera localization and provides a complete classification for the topic. “Overview” section presents an overview of image-based camera localization and is mapped as a tree structure. “Reviews on image-based camera localization” section introduces each aspect of the classification. “Discussion” section presents discussions and analyzes trends of future developments “Conclusion” section makes a conclusion of the paper.

## Overview

What is image-based camera localization? Image-based camera localization is to compute camera poses under a world coordinate system from images or videos captured by the cameras. Based on whether the environment is known beforehand or not, image-based camera localization can be classified into two categories: one with known environment and the other with unknown environment.

Let *n* be the number of points used. The approach with known environments consists of methods with 3 ≤ *n* < 6 and methods with *n* ≥ 6. These are PnP problems. In general, the problems with 3 ≤ *n* < 6 are nonlinear and those with *n* ≥ 6 are linear.

**Fig. 1:**
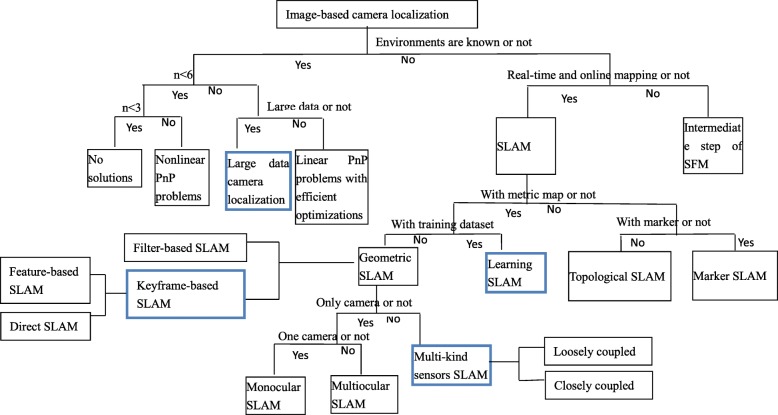
Overview of image-based camera localization

The approach with unknown environments can be divided into methods with online and real-time environment mapping and those without online and real-time environment mapping. The former is the commonly known Simultaneous Localization and Mapping (SLAM) and the latter is an intermediate procedure of the commonly known structure from motion (SFM). According to different map generations, SLAM is divided into four parts: geometric metric SLAM, learning SLAM, topological SLAM, and marker SLAM. Learning SLAM is a new research direction recently. We think it is different from geometric metric SLAM and topological SLAM by a single category. Learning SLAM can obtain camera pose and 3D map but needs a prior dataset to train the network. The performance of learning SLAM depends on the used dataset to a great extent and it has low generalization capability. Therefore, learning SLAM is not as flexible as geometric metric SLAM and its obtained 3D map outside the used dataset is not as accurate as geometric metric SLAM most of the time. However, simultaneously, learning SLAM has a 3D map other than topology representations. Marker SLAM computes camera poses from known structured markers without knowing the complete environment. Geometric metric SLAM consists of monocular SLAM, multiocular SLAM, and multi-kind sensor SLAM. Moreover, geometric metric SLAM can be classified into filter-based SLAM and keyframe-based SLAM. Keyframe-based SLAM can be further divided into feature-based SLAM and direct SLAM. Multi-kind sensors SLAM can be divided into loosely coupled SLAM and closely coupled SLAM. These classifications of image-based camera localization methods are visualized as a logical tree structure, as shown in Fig. 1, where current active topics are indicated with bold borders. We think that these topics are camera localization from large data, learning SLAM, keyframe-based SLAM, and multi-kind sensors SLAM.

## Reviews on image-based camera localization

### Known environments

Camera pose determination from known 3D space points is called the perspective-n-point problem, namely, the PnP problem. When *n* = 1,2, there are no solutions for PnP problems because they are under constraints. When *n* ≥ 6, PnP problems are linear. When *n* = 3, 4, 5, the original equations of PnP problems are usually nonlinear. The PnP problem dated from 1841 to 1903. Grunert [5], Finsterwalder to Scheufele [6] concluded that the P3P problem has at most four solutions and the P4P problem has a unique solution in general. The PnP problem is also the key relocalization for SLAM.

#### PnP problems with *n* = 3, 4, 5

The methods to solve PnP problems with *n* = 3, 4, 5 focus on two aspects. One aspect studies the solution numbers or multisolution geometric configuration of the nonlinear problems. The other aspect studies eliminations or other solving methods for camera poses.

The methods that focus on the first aspect are as follows. Grunert [5], Finsterwalder and Scheufele [6] pointed out that P3P has up to four solutions and P4P has a unique solution. Fischler and Bolles [7] studied P3P for RANSAC of PnP and found that four solutions of P3P are attainable. Wolfe et al. [8] showed that P3P mostly has two solutions; they determined the two solutions and provided the geometric explanations that P3P can have two, three, or four solutions. Hu and Wu [9] defined distance-based and transformation-based P4P problems. They found that the two defined P4P problems are not equivalent; they found that the transformation-based problem has up to four solutions and distance-based problem has up to five solutions. Zhang and Hu [10] provided sufficient and necessary conditions in which P3P has four solutions. Wu and Hu [11] proved that distance-based problems are equivalent to rotation-transformation-based problems for P3P and distance-based problems are equivalent to orthogonal-transformation-based problems for P4P/P5P. In addition, they showed that for any three non-collinear points, the optical center can always be found such that the P3P problem formed by these three control points and the optical center will have four solutions, which is its upper bound. Additionally, a geometric approach is provided to construct these four solutions. Vynnycky and Kanev [12] studied the multisolution probabilities of the equilateral P3P problem.

The methods that focus on the second aspect of PnP problems with *n* = 3, 4, 5 are as follows. Horaud et al. [13] described an elimination method for the P4P problem to obtain a unitary quartic equation. Haralick et al. [14] reviewed six methods for the P3P problem, which are [5–7, 15–17]. Dementhon and Davis [18] presented a solution of the P3P problem by an inquiry table of quasi-perspective imaging. Quan and Lan [19] linearly solved the P4P and P5P problems. Gao et al. [20] used Wu’s elimination method to obtain complete solutions of the P3P problem. Wu and Hu [10] introduced a depth-ratio-based approach to represent the solutions of the complete PnP problem. Josephson and Byrod [21] used Grobner bases method to solve the P4P problem for radial distortion of camera with unknown focal length. Hesch et al. [22] studied nonlinear square solutions of PnP with *n* >= 3. Kneip et al. [23] directly solved the rotation and translation solutions of the P3P problem. Kneip et al. [24] presented a unified PnP solution that can deal with generalized cameras and multisolutions with global optimizations and linear complexity. Kuang and Astrom [25] studied the PnP problem with unknown focal length using points and lines. Z. Kukelova et al. [26] studied the PnP problem with unknown focal length for images with radial distortion. Ventura et al. [27] presented a minimal solution to the generalized pose-and-scale problem. Zheng et al. [28] introduced an angle constraint and derived a compact bivariate polynomial equation for each P3P and then proposed a general method for the PnP problem with unknown focal length using iterations. Later, Zheng and Kneip [29] improved their work without requiring point order and iterations. Wu [30] studied PnP solutions with unknown focal length and *n* = 3.5. Albl et al. [31] studied the pose solution of a rolling shutter camera and improved the result later in 2016.

#### PnP problems with *n* ≥ 6

When *n*> = 6, PnP problems are linear and studies on them focus on two aspects. One aspect studies efficient optimizations for camera poses from smaller number of points. The other aspect studies fast camera localization from large data.

The studies on the first aspect are as follows. Lu et al. [32] gave a global convergence algorithm using collinear points. Schweighofer and Pinz [33] studied multisolutions of a planar target. Wu et al. [34] presented invariant relationships between scenes and images, and then a robust RANSAC PNP using the invariants. Lepetit et al. [35] provided an accurate O(n) solution to the PnP problem, called EPnP, which is widely used today. The pose problem of a rolling shutter camera was studied in [36] with bundle adjustments. A similar problem was also studied in [37] using B-spline covariance matrix. Zheng et al. [38] used quaternion and Grobner bases to provide a global optimized solution of the PnP problem. A very fast solution to the PnP Problem with algebraic outlier rejection was given in [39]. Svarm et al. [40] studied accurate localization and pose estimation for large 3D models considering gravitational direction. Ozyesil et al. [41] provided robust camera location estimation by convex programming. Brachmann et al. [42] showed uncertainty-driven 6D pose estimation of objects and scenes from a single RGB image. Feng et al. [43] proposed a hand-eye calibration-free strategy to actively relocate a camera in the same 6D pose by sequentially correcting relative 3D rotation and translation. Nakano [44] solved three kinds of PnP problems by the Grobner method: PnP problem for calibrated camera, PnPf problem for cameras with unknown focal length, PnPfr problem for cameras with unknown focal length and unknown radial distortions.

The studies on the second aspect that focus on fast camera localization from large data are as follows. Arth et al. [45, 46] presented real-time camera localizations for mobile phones. Sattler et al. [47] derived a direct matching framework based on visual vocabulary quantization and a prioritized correspondence search with known large-scale 3D models of urban scenes. Later, they improved the method by active correspondence search in [48]. Li et al. [49] devised an adaptive, prioritized algorithm for matching a representative set of SIFT features covering a large scene to a query image for efficient localization. Later Li et al. [50] provided a full 6-DOF-plus-intrinsic camera pose with respect to a large geo-registered 3D point cloud. Lei et al. [51] studied efficient camera localization from street views using PCA-based point grouping. Bansal and Daniilidis [52] proposed a purely geometric correspondence-free approach to urban geo-localization using 3D point-ray features extracted from the digital elevation map of an urban environment. Kendall et al. [53] presented a robust and real-time monocular 6-DOF relocalization system by training a convolutional neural network (CNN) to regress the 6-DOF camera pose from a single RGB image in an end-to-end manner. Wang et al. [54] proposed a novel approach to localization in very large indoor spaces that takes a single image and a floor plan of the environment as input. Zeisl et al. [55] proposed a voting-based pose estimation strategy that exhibits O(n) complexity in terms of the number of matches and thus facilitates considering more number of matches. Lu et al. [56] used a 3D model reconstructed by a short video as the query to realize 3D-to-3D localization under a multi-task point retrieval framework. Valentin et al. [57] trained a regression forest to predict mixtures of anisotropic 3D Gaussians and showed how the predicted uncertainties can be taken into account for continuous pose optimization. Straub et al. [58] proposed a relocalization system that enables real-time, 6D pose recovery for wide baselines by using binary feature descriptors and nearest-neighbor search of locality sensitive hashing. Feng et al. [59] achieved fast localization in large-scale environments by using supervised indexing of binary features, where randomized trees were constructed in a supervised training process by exploiting the label information derived from multiple features that correspond to a common 3D point. Ventura and Höllerer [60] proposed a system of arbitrary wide-area environments for real-time tracking with a handheld device. The combination of a keyframe-based monocular SLAM system and a global localization method was presented in [61]. A book of large-scale visual geo-localization was published in [62]. Liu et al. [63] showed efficient global 2D-3D matching for camera localization in a large-scale 3D map. Campbell [64] presented a method for globally optimal inlier set maximization for simultaneous camera pose and feature correspondence. Real-time SLAM relocalization with online learning of binary feature indexing was proposed by [65]. Wu et al. [66] proposed CNNs for camera relocalization. Kendall and Cipolla [67] explored a number of novel loss functions for learning camera poses, which are based on geometry and scene reprojection error. Qin et al. [68] developed a method of relocalization for monocular visual-inertial SLAM. Piasco et al. [4] presented a survey on visual-based localization from heterogeneous data. A geometry-based point cloud reduction method for mobile augmented reality system was presented in [69].

From above the studies for known environments, we see that fast camera localization from large data has attracted more and more attention. This is because there are many applications of camera localization for large data, for example, location-based services, relocalization of SLAM for all types of robots, and AR navigations.

### Unknown environments

Unknown environments can be reconstructed from videos in real time and online. Simultaneously, camera poses are computed in real time and online. These are the commonly known SLAM technologies. If unknown environments are reconstructed from multiview images without requiring speed and online computation, it is the known SFM, in which solving for the camera pose is an intermediate step and not the final aim; therefore, we only mention few studies on SFM, and do not provide an in-depth overview in the following. Studies on SLAM will be introduced in detail.

#### SLAM

SLAM was dated from 1986 in the study [70]: “On the representation and estimation of spatial uncertainty,” published in the International Journal of Robotics Research. In 1995, the acronym SLAM was then coined in the study [71]: “Localisation of automatic guided vehicles,” 7th International Symposium on Robotics Research. According to different map generations, the studies on SLAM can be divided into four categories: geometric metric SLAM, learning SLAM, topological SLAM, and marker SLAM. Due to its accurate computations, geometric metric SLAM has attracted increasing attention. Learning SLAM is a new topic gaining attention due to the development of deep learning. Studies on pure topological SLAM are decreasing. Marker SLAM is more accurate and stable. There is a study in [2] that reviews recent advances of SLAM covering a broad set of topics including robustness and scalability in long-term mapping, metric and semantic representations for mapping, theoretical performance guarantees, active SLAM, and exploration. In the following, we introduce geometric metric SLAM, learning SLAM, topological SLAM, and marker SLAM.

A. Geometric metric SLAM
  Geometric metric SLAM computes 3D maps with accurate mathematical equations. Based on the different sensors used, geometric metric SLAM is divided into monocular SLAM, multiocular SLAM, and multi-kind sensors SLAM. Based on the different techniques used, geometric metric SLAM is divided into filter-based SLAM and keyframe-based SLAM and also, there is another class of SLAM: grid-based SLAM, of which minority deal with images and most deal with laser data. Recently, there was a review on keyframe-based monocular SLAM, which provided in-depth analyses [3].
  1. Monocular SLAM
    A.1.1) Filter-based SLAM
      One part of monocular SLAM is the filter-based methods. The first one is the Mono-SLAM proposed by Davison [72] based on extended Kalman filter (EKF). Later, the work was developed by them further in [73, 74]. Montemerlo and Thrun [75] proposed monocular SLAM based on a particle filter. Strasdat et al. [76, 77] discussed why filter-based SLAM is used by comparing filter-based and keyframe-based methods. The conference paper in ICRA 2010 by [76] received the best paper award, where they pointed out that keyframe-based SLAM can provide more accurate results. Nuchter et al. [78] used a particle filter for SLAM to map large 3D outdoor environments. Huang et al. [79] addressed two key limitations of the unscented Kalman filter (UKF) when applied to the SLAM problem: the cubic computational complexity in the number of states and the inconsistency of the state estimates. They introduced a new sampling strategy for the UKF, which has constant computational complexity, and proposed a new algorithm to ensure that the unobservable subspace of the UKF’s linear-regression-based system model has the same dimension as that of the nonlinear SLAM system. Younes et al. [3] also stated that filter-based SLAM was common before 2010 and most solutions thereafter designed their systems around a non-filter, keyframe-based architecture.
    A.1.2) Keyframe-based SLAM
      The second part of monocular SLAM is the keyframe-based methods. Keyframe-based SLAM can be further categorized into: feature-based methods and direct methods. a) Feature-based SLAM: The first keyframe-based feature SLAM was PTAM proposed in [80]. Later the method was extended to combine edges in [81] and extended to a mobile phone platform by them in [82]. The keyframe selections were studied in [83, 84]. SLAM++ with loop detection and object recognition was proposed in [85]. Dynamic scene detection and adapting RANSAC was studied by [86]. Regarding dynamic objects, Feng et al. [87] proposed a 3D-aided optical flow SLAM. ORB SLAM [88] can deal with loop detection, dynamic scene detection, monocular, binocular, and deep images. The method of [89] can run in a large-scale environment using submap and linear program to remove outlier. b) Direct SLAM: The second part of monocular SLAM is the direct method. Newcombe et al. [90] proposed DTAM, the first direct SLAM, where detailed textured dense depth maps, at selected keyframes, are produced and meanwhile camera pose is tracked at frame rate by entire image alignment against the dense textured model. A semi-dense visual odometry (VO) was proposed in [91]. LSD SLAM by [92] provided a dense SLAM suitable for large-scale environments. Pascoe et al. [93] proposed a direct dense SLAM for road environments for LIDAR and cameras. A semi VO on a mobile phone was performed by [94].
  2. Multiocular SLAM
    Multiocular SLAM uses multiple cameras to compute camera poses and 3D maps. Most of the studies focus on binocular vision. They are also the bases of multiocular vision.
    Konolige and Agrawal [95] matched visual frames with large numbers of point features using classic bundle adjustment techniques but kept only relative frame pose information. Mei et al. [96] used local estimation of motion and structure provided by a stereo pair to represent the environment in terms of a sequence of relative locations. Zou and Tan [97] studied SLAM of multiple moving cameras in which a global map is built. Engle et al. [98] proposed a novel large-scale direct SLAM algorithm for stereo cameras. Pire et al. [99] proposed a stereo SLAM system called S-PTAM that can compute the real scale of a map and overcome the limitation of PTAM for robot navigation. Moreno et al. [100] proposed a novel approach called sparser relative bundle adjustment (SRBA) for a stereo SLAM system. Artal and Tardos [101] presented ORB-SLAM2 which is a complete SLAM system for monocular, stereo, and RGB-D cameras, with map reuse, loop closing, and relocalization capabilities. Zhang et al. [102] presented a graph-based stereo SLAM system using straight lines as features. Gomez-Ojeda et al. [103] proposed PL-SLAM, a stereo visual SLAM system that combines both points and line segments to work robustly in a wider variety of scenarios, particularly in those where point features are scarce or not well-distributed in an image. A novel direct visual-inertial odometry method for stereo cameras was proposed in [104]. Wang et al. [105] proposed stereo direct sparse odometry (Stereo DSO) for highly accurate real-time visual odometry estimation of large-scale environments from stereo cameras. Semi-direct visual odometry (SVO) for monocular and multi-camera systems was proposed in [106]. Sun et al. [107] proposed a stereo multi-state constraint Kalman filter (S-MSCKF). Compared with multi-state constraint Kalman filter (MSCKF), S-MSCKF exhibits significantly greater robustness.
    Multiocular SLAM has higher reliability than monocular SLAM. In general, multiocular SLAM is preferred if hardware platforms are allowed.
  3. Multi-kind sensors SLAM
    Here, multi-kind sensors are limited to vision and inertial measurement unit (IMU); other sensors are not introduced here. This is because, recently, vision and IMU fusion has attracted more attention than others.
    In robotics, there are many studies on SLAM that combine cameras and IMU. It is common for mobile devices to be equipped with a camera and an inertial unit. Cameras can provide rich information of a scene. IMU can provide self-motion information and also provide accurate short-term motion estimates at high frequency. Cameras and IMU have been thought to be complementary of each other. Because of universality and complementarity of visual-inertial sensors, visual-inertial fusion has been a very active research topic in recent years. The main research approaches on visual-inertial fusion can be divided into two categories, namely, loosely coupled and tightly coupled approaches.
    A.3.1) Loosely coupled SLAM In loosely coupled systems, all sensor states are independently estimated and optimized. Integrated IMU data are incorporated as independent measurements in stereo vision optimization in [108]. Vision-only pose estimates are used to update an EKF so that IMU propagation can be performed [109]. An evaluation of different direct methods for computing frame-to-frame motion estimates of a moving sensor rig composed of an RGB-D camera and an inertial measurement unit is given and the pose from visual odometry is added to the IMU optimization frame directly in [110].
    A.3.2) Tightly coupled SLAM In tightly coupled systems, all sensor states are jointly estimated and optimized. There are two approaches for this, namely, filter-based and keyframe nonlinear optimization-based approaches.
      - Filter-based approach The filter-based approach uses EKF to propagate and update motion states of visual-inertial sensors. MSCKF in [111] uses an IMU to propagate the motion estimation of a vehicle and update this motion estimation by observing salient features from a monocular camera. Li and Mourikis [112] improved MSCKF, by proposing a real-time EKF-based VIO algorithm, MSCKF2.0. This algorithm can achieve consistent estimation by ensuring correct observability properties of its linearized system model and performing online estimation of the camera-to-inertial measurement unit calibration parameters. Li et al. [113], Li and Mourikis [114] implemented real-time motion tracking on a cellphone using inertial sensing and a rolling-shutter camera. MSCKF algorithm is the core algorithm of Google’s Project Tango https://get.google.com/tango/. Clement et al. [115] compared two modern approaches: MSCKF and sliding window filter (SWF). SWF is more accurate and less sensitive to tuning parameters than MSCKF. However, MSCKF is computationally cheaper, has good consistency, and improves accuracies because more features are tracked. Bloesch et al. [116] presented a monocular visual inertial odometry algorithm by directly using pixel intensity errors of image patches. In this algorithm, by directly using the intensity errors as an innovation term, the tracking of multilevel patch features is closely coupled to the underlying EKF during the update step.
      - Keyframe nonlinear optimization-based approach The nonlinear optimization-based approach uses keyframe-based nonlinear optimization, which may potentially achieve higher accuracy due to the capability to limit linearization errors through repeated linearization of the inherently nonlinear problem. Forster et al. [117] presented a preintegration theory that appropriately addresses the manifold structure of the rotation group. Moreover, it is shown that the preintegration IMU model can be seamlessly integrated into a visual-inertial pipeline under the unifying framework of factor graphs. The method is short for GTSAM. Leutenegger et al. [118] presented a novel approach, OKVIS, to tightly integrate visual measurements with IMU measurements, where a joint nonlinear cost function that integrates an IMU error term with the landmark reprojection error in a fully probabilistic manner is optimized. Moreover, to ensure real-time operation, old states are marginalized to maintain a bounded-sized optimization window. Li et al. [119] proposed tightly coupled, optimization-based, monocular visual-inertial state estimation for camera localization in complex environments. This method can run on mobile devices with a lightweight loop closure. Following ORB monocular SLAM [88], a tightly coupled visual-inertial slam system was proposed in [120].
        In loosely coupled systems, it is easy to process frame and IMU data. However, in tightly coupled systems, to optimize all sensor states jointly, it is difficult to process frame and IMU data. In terms of estimation accuracy, tightly coupled methods are more accurate and robust than loosely coupled methods. Tightly coupled methods have become increasingly popular and have attracted great attention by researchers.
B. Learning SLAM
  Learning SLAM is a new topic that gained attention recently due to the development of deep learning. We think it is different from geometric metric SLAM and topological SLAM by a single category. Learning SLAM can obtain camera pose and 3D map but needs a prior dataset to train the network. The performance of learning SLAM depends on the used dataset greatly and it has low generalization ability. Therefore, learning SLAM is not as flexible as geometric metric SLAM and the geometric map obtained outside the used dataset is not as accurate as geometric metric SLAM most of the time. However, simultaneously, learning SLAM has a 3D map other than 2D graph representations.
  Tateno et al. [121] used CNNs to predict dense depth maps and then used keyframe-based 3D metric direct SLAM to compute camera poses. Ummenhofer et al. [122] trained multiple stacked encoder-decoder networks to compute depth and camera motion from successive, unconstrained image pairs. Vijayanarasimhan et al. [123] proposed a geometry-aware neural network for motion estimation in videos. Zhou et al. [124] presented an unsupervised learning framework for estimating monocular depth and camera motion from video sequences. Li et al. [125] proposed a monocular visual odometry system using unsupervised deep learning; they used stereo image pairs to recover the scales. Clark et al. [126] presented an on-manifold sequence-to-sequence learning approach for motion estimation using visual and inertial sensors. Detone et al. [127] presented a point tracking system powered by two deep CNNs, MagicPoint and MagicWarp. Gao and Zhang [128] presented a method for loop closure detection based on the stacked denoising auto-encoder. Araujo et al. [129] proposed a recurrent CNN-based visual odometry approach for endoscopic capsule robots.
  Learning SLAM increases gradually these years. However, due to lower speed and generalization capabilities of the learning methods, using geometric methods is still centered for practical applications.
C. Topological SLAM
  Topological SLAM does not need accurate computation of 3D maps and represents the environment by connectivity or topology. Kuipers and Byun [130] used a hierarchical description of the spatial environment, where a topological network description mediates between a control and metrical level; moreover, distinctive places and paths are defined by their properties at the control level and serve as nodes and arcs of the topological model. Ulrich and Nourbakhsh [131] presented an appearance-based place recognition system for topological localization. Choset and Nagatani [132] exploited the topology of a robot’s free space to localize the robot on a partially constructed map and the topology of the environment was encoded in a generalized Voronoi graph. Kuipers et al. [133] described how a local perceptual map can be analyzed to identify a local topology description and abstracted to a topological place. Chang et al. [134] presented a prediction-based SLAM algorithm to predict the structure inside an unexplored region. Blanco et al. [135] used Bayesian filtering to provide a probabilistic estimation based on the reconstruction of a robot path in a hybrid discrete-continuous state space. Blanco et al. [136] presented spectral graph partitioning techniques for the automatic generation of sub-maps. Kawewong et al. [137] proposed dictionary management to eliminate redundant search for indoor loop-closure detection based on PIRF extraction. Sünderhauf and Protzel [138] presented a back-end formulation for SLAM using switchable constraints to recognize and reject outliers during loop-closure detection by making the topology of the underlying factor graph representation. Latif et al. [139] described a consensus-based approach for robust place recognition to detect and remove past incorrect loop closures to deal with the problem of corrupt map estimates. Latif et al. [140] presented a comparative analysis for graph SLAM, where graph nodes are camera poses connected by odometry or place recognition. Vallvé et al. [141] proposed two simple algorithms for SLAM sparsification, factor descent and non-cyclic factor descent.
  As shown in some above mentioned works, topological SLAM has been modified into metric SLAM as loop detection these years. Studies on pure topological SLAM are reducing.
D. Marker SLAM
  We introduced studies on image-based camera localization for both known and unknown environments above. In addition, there are some studies to localize cameras using some prior environment knowledge, but not a 3D map such as markers. These works are considered to be with semi-known environments.
  In 1991, Gatrell et al. [142] designed a concentric circular marker, which was modified with additional color and scale information in [143]. Ring information was considered in the marker by [144]. Kato and Billinghurst [145] presented the first augmented reality system based on fiducial markers known as the ARToolkit, where the marker used is a black enclosed rectangle with simple graphics or text. Naimark and Foxlin [146] developed a more general marker generation method, which encodes a bar code into a black circular region to produce more markers. A square marker was presented by [147]. Four circles at the corners of a square were proposed by [148]. A black rectangle enclosed with black and white blocks known as the ARTag was proposed by [149, 150]. From four marker points, Maidi et al. [151] developed a hybrid approach that combines an iterative method based on the EKF and an analytical method with direct resolution of pose parameter computation. Recently, Bergamasco et al. [152] provided a set of circular high-contrast dots arranged in concentric layers. DeGol et al. [153] introduced a fiducial marker, ChromaTag, and a detection algorithm to use opponent colors to limit and reject initial false detections and grayscale. Munoz-Salinas et al. [154] proposed to detect key points for the problem of mapping and localization from a large set of squared planar markers. Eade and Drummond [155] proposed real-time global graph SLAM for sequences with several hundreds of landmarks. Wu [156] studied a new marker for camera localization that does not need matching.

#### SFM

In SFM, camera pose computation is only an intermediate step. Therefore, in the following, we give a brief introduction of camera localization SFM.

In the early stages of SFM development, there were more studies on relative pose solving. One of the useful studies is the algorithm for five-point relative pose problem in [157], which has less degeneracies than other relative pose solvers. Lee et al. [158] studied relative pose estimation for a multi-camera system with known vertical direction. Kneip and Li [159] presented a novel solution to compute the relative pose of a generalized camera. Chatterjee and Govindu [160] presented efficient and robust large-scale averaging of relative 3D rotations. Ventura et al. [161] proposed an efficient method for estimating the relative motion of a multi-camera rig from a minimal set of feature correspondences. Fredriksson et al. [162] estimated the relative translation between two cameras and simultaneously maximized the number of inlier correspondences.

Global pose studies are as follows. Park et al. [163] estimated the camera direction of a geotagged image using reference images. Carlone et al. [164] surveyed techniques for 3D rotation estimation. Jiang et al. [165] presented a global linear method for camera pose registration. Later, the method was improved by [166] and [167].

Recently, hybrid incremental and global SFM have been developed. Cui et al. [168, 169], estimated rotations by a global method and translations by an incremental method and proposed community-based SFM. Zhu et al. [170] presented parallel SFM from local increment to global averaging.

A recent survey on SFM is presented in [171]. In addition, there are some studies on learning depth from a single image. From binoculars, usually disparity maps are learnt. Please refer to the related works ranked in the website of KITTI dataset.

## Discussion

From the above techniques, we can see that currently there are less and less studies on the PnP problem in a small-scale environment. Similarly, there are few studies on SFM using traditional geometric methods. However, for SLAM, both traditional geometric and learning methods are still popular.

Studies that use deep learning for image-based camera localization are increasing gradually. However, in practical applications, using geometric methods is still centered. Deep learning methods can provide efficient image features and compensate for geometric methods.

The PnP problem or relocalization of SLAM in a large-scale environment has not been solved well and deserves further research. For reliability and low cost practical applications, multi low cost sensor fusion for localization but vision sensor centered is an effective way.

In addition, some works study the pose problem of other camera sensors, such as the epipolar geometry of a rolling shutter camera in [172, 173] and radial-distorted rolling-shutter direct SLAM in [174]. Gallego et al. [175], Vidal et al. [176], Rebecq et al. [177] studied event camera SLAM.

With the increasing development of SLAM, maybe it starts the age of embedded SLAM algorithms as shown by [178]. We think integrating the merits of all kinds of techniques is a trend for a practical SLAM system, such as geometric and learning fusion, multi-sensor fusion, multi-feature fusion, feature based and direct approaches fusion. Integration of these techniques may solve the current challenging difficulties such as poorly textured scenes, large illumination changes, repetitive textures, and highly dynamic motions.

## Conclusion

Image-based camera localization has important applications in fields such as virtual reality, augmented reality, robots. With the rapid development of artificial intelligence, these fields have become high-growth markets, and are attracting much attention from both academic and industrial communities.

We presented an overview of image-based camera localization, in which a complete classification is provided. Each classification is further divided into categories and the related works are presented along with some analyses. Simultaneously, the overview is described in a tree structure, as shown in Fig. 1. In the tree structure, the current popular topics are denoted with bold blue borders. These topics include large data camera localization, learning SLAM, multi-kind sensors SLAM, and keyframe-based SLAM. Future developments were also discussed in the Discussion section.
